# Direct targets of pSTAT5 signalling in erythropoiesis

**DOI:** 10.1371/journal.pone.0180922

**Published:** 2017-07-21

**Authors:** Kevin R. Gillinder, Hugh Tuckey, Charles C. Bell, Graham W. Magor, Stephen Huang, Melissa D. Ilsley, Andrew C. Perkins

**Affiliations:** 1 Cancer Genomics Group, Mater Research Institute - University of Queensland, Translational Research Institute, Woolloongabba, Brisbane, Queensland, Australia; 2 Faculty of Medicine and Biomedical Sciences, University of Queensland, St. Lucia, Brisbane, Queensland, Australia; 3 Princess Alexandra Hospital, Brisbane, Queensland, Australia; Emory University, UNITED STATES

## Abstract

Erythropoietin (EPO) acts through the dimeric erythropoietin receptor to stimulate proliferation, survival, differentiation and enucleation of erythroid progenitor cells. We undertook two complimentary approaches to find EPO-dependent pSTAT5 target genes in murine erythroid cells: RNA-seq of newly transcribed (4sU-labelled) RNA, and ChIP-seq for pSTAT5 30 minutes after EPO stimulation. We found 302 pSTAT5-occupied sites: ~15% of these reside in promoters while the rest reside within intronic enhancers or intergenic regions, some >100kb from the nearest TSS. The majority of pSTAT5 peaks contain a central palindromic GAS element, TTCYXRGAA. There was significant enrichment for GATA motifs and CACCC-box motifs within the neighbourhood of pSTAT5-bound peaks, and GATA1 and/or KLF1 co-occupancy at many sites. Using 4sU-RNA-seq we determined the EPO-induced transcriptome and validated differentially expressed genes using dynamic CAGE data and qRT-PCR. We identified known direct pSTAT5 target genes such as *Bcl2l1*, *Pim1* and *Cish*, and many new targets likely to be involved in driving erythroid cell differentiation including those involved in mRNA splicing (*Rbm25*), epigenetic regulation (*Suv420h2*), and EpoR turnover (*Clint1/EpsinR*). Some of these new EpoR-JAK2-pSTAT5 target genes could be used as biomarkers for monitoring disease activity in polycythaemia vera, and for monitoring responses to JAK inhibitors.

## Introduction

EPO is produced by specialised cells in the kidney in response to reduced oxygen tension. It binds to the erythropoietin receptor (EpoR) expressed on erythroid progenitor cells to induce proliferation, differentiation, survival and enucleation. Thus, an EPO-EpoR feedback loop re-establishes physiologically appropriate numbers of circulating mature red blood cells in response to need. Exogenous use of EPO, inherited mutations in the EpoR [1], and acquired gain-of-function mutations in JAK2 [2,3], induce supra-physiological production of red blood cells. Conversely, loss-of-function experiments have shown both EPO and EpoR are essential for the proliferation and survival of erythroid cells [4,5].

There are differing models of EPO-EpoR signalling. One model suggests EPO induces dimerization of the EpoR and this is essential for signaling [6]. Others suggest EpoR exists as pre-assembled homodimer complexed with JAK2 bound to the box1-2 motif in the cytoplasmic membrane-proximal domain of each monomer. Binding of EPO causes a conformational twist in the orientation of the two EpoR monomers such that cross-inhibition of JAK2 kinase domains (via the pseudo-kinase domains) is released, resulting in JAK2 phosphorylation [7]. This mechanism of signal propagation applies to all type 1 dimeric cytokine receptors [8]. Activating mutations in the pseudo-kinase domain of JAK2, such as JAK2V617F, lead to ligand-independent phosphorylation of JAK2 and downstream activation of signalling pathways in patients with polycythemia vera (PV). STAT5 is recruited to pY343 of the EpoR via its SH2 domain [9], where it is phosphorylated by JAK2. This results in dimerization, translocation to the nucleus, and DNA binding to palindromic gamma-activated sequences or ‘GAS′ motifs (TTCYXRGAA) in promoters and enhancers; it binds as a dimer [10] or a tetramer [11]. Thus, EPO induces changes in gene expression through the JAK2-pSTAT5 pathway. There are duplicated STAT5 genes, *Stat5a* and *Stat5b*, which have 95% amino acid sequence identity [12]. They play specific roles in responses to various hormones in non-erythroid cells [13,14], but they play redundant roles in erythropoiesis; i.e. *Stat5a-/- Stat5b-/-* mice die *in utero* from anemia [15]. The phenotype is like *Epo*, *EpoR* and *Jak2* gene knockout mice [4,5].

Activated pJAK2 phosphorylates additional tyrosine (pY) residues in the cytoplasmic tail of the EpoR which leads to engagement of other SH2 domain-containing cytoplasmic signalling molecules. For example, pY429/Y431 and pY479 bind the p85 subunit of PI3 kinase leading to subsequent engagement of p110 and phosphorylation of downstream transcription factors (TFs). Gain-of-function mutations in p85 lead to constitutive EpoR activity whereas p85 knockout mice display anemia [16]. The raf-MAPK and LYN kinase pathways are also activated in erythroid cells by EPO [17]. A truncated EpoR containing the binding site for pSTAT5 (Y343), but missing C-terminal Y residues, is sufficient to rescue responses to anemic stress *in vivo* [9], suggesting STAT5 engagement is critical.

The *Bcl2l1* gene is a known direct target of pSTAT5 in erythroid cells [15], and it is required for a pro-survival signal in response to EPO [18]. It has a long second intron containing enhancers, some of which have been shown to bind STAT1 or STAT5 [15,19], and some of which bind other erythroid TFs such as GATA1 and KLF1 [20,21]. Furthermore, *Bcl2l1* expression is dependent on both GATA1 and KLF1 [22,23]. The *Bcl2l1* gene undergoes dynamic alternative splicing during erythroid differentiation leading to the mutually exclusive production of short (Bcl-x_S_) and long (Bcl-x_L_) protein isoforms. The long isoform is a pro-survival factor while the short isoform is pro-apoptotic [24].

Other well-studied targets of EpoR signalling include members of suppressor-of-cytokine-signalling (SOCS) gene family, particularly *Socs3* and *Cish* [25,26]. The protein products of these genes are responsible for rapid down-regulation of the EPO-EpoR complex via engagement of ubiquitin ligase pathways, receptor internalisation and its degradation in the proteasome and lysosome pathways [27]. Thus, SOCS proteins rapidly dampen EPO-induced signals. There are likely many other direct targets of pSTAT5 in erythroid cells but positive identification has been hampered by the lack of ChIP-seq datasets. We have undertaken the first ChIP-seq for pSTAT5 in erythroid cells in response to EPO and found 302 robust sites of genome occupancy. While some pSTAT5 is bound at promoters, the majority occupies enhancers, often in concert with GATA1 and KLF1. Those sites not bound by these TFs tend to be bound by STAT5 in other cell types suggesting generic targets and functions for STAT5 in many cells. We used 4sU-RNA-labelling to determine rapidly induced genes and also examined the dynamics of gene induction in response to EPO using qRT-PCR and published dynamic CAGE data [28]. We found expected direct targets of EpoR-JAK2-pSTAT5 signalling such as *Cish* and *Bcl2l1*, but also several novel target genes with likely roles in EpoR turnover, chromatin compaction, alternative splicing and other processes critical to terminal erythroid differentiation. This work provides new insights into how EPO works and provides a list of possible biomarkers to monitor disease activity in PV.

## Results

### Direct targets of pSTAT5 in erythroid cells

The murine erythroid cell line, J2E, expresses ~1000 copies of the EpoR on the cell surface and undergoes terminal erythroid differentiation in response to EPO. J2E have been employed by the FANTOM5 consortium for high resolution mapping of dynamic changes in CAGE tags at erythroid promoters and enhancers in response EPO [28]. They are immortalised at the pro-erythroblast stage like MEL cells, G1-ER cells [23], and K1-ER cells [29]. Thus, data sets such as RNA-seq, histone ChIP-seq and TF ChIP-seq generated in these cells can be employed to interrogate the genomic landscape of J2E.

**Fig 1:**
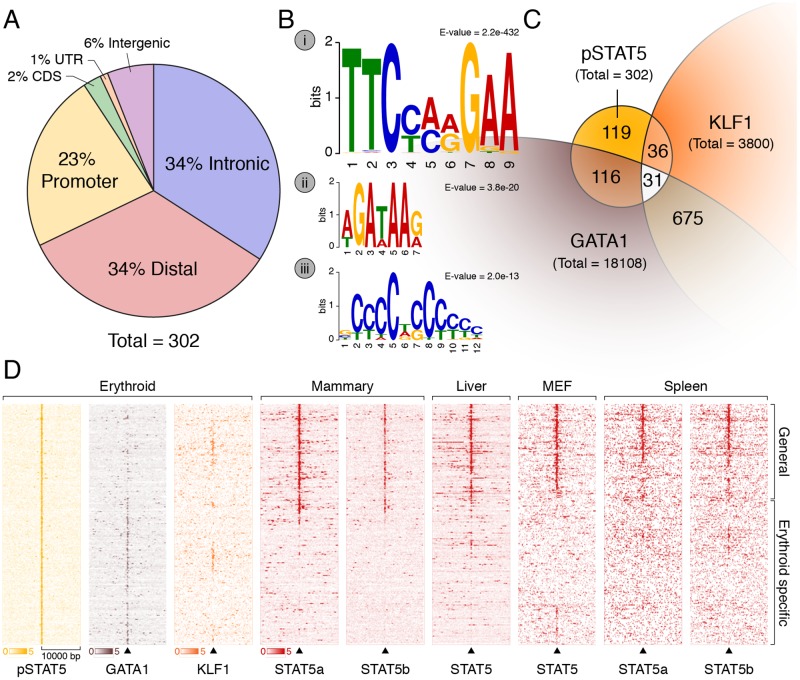
pSTAT5 binds promoters and enhancers of key erythroid genes in concert with GATA1 and KLF1. (A) 302 peaks were annotated with respect to the nearest TSS as defined by RefSeq. Only ~23% of peaks reside within gene promoters (1 kb upstream or 100 nt downstream of a TSS). The majority are intronic or distal (within 50 kb of a gene body) consistent with enhancer occupancy. (B) *De novo* motif discovery on all 302 peaks using MEME (see Methods) identified highly significant enrichment of a (i) palindromic GAS element (TTCYMRGAA), a (ii) GATA binding site (WGATAR), and a (iii) KLF binding site (CCMCRCCCN). No other significantly enriched motifs were found. (C) Venn diagram and of erythroid co-occupancy between pSTAT5, KLF1 and GATA1 at key erythroid enhancers and promoters. KLF1 ChIP-seq was generated in K1-ER cells and GATA1 ChIP-seq was generated in G1-ER4 cells after induction with tamoxifen. Co-occupancy defined as ChIP summits within 500 bp. (D) Density heat-map of ChIP signal centred on pSTAT5 peak summits from GATA1 and KLF1 as above (C) and additional STAT5 ChIP in other murine tissues. The Y-axis represents individual peak regions, and the X-axis represents 10 kb surrounding the summit. Read intensities were normalized to number of reads in dataset and hierarchically clustered according to intensity within 500 bp of peak centre.

After EPO stimulation (10U/ml) for 30 min (S1A Fig), pSTAT5 was robustly induced in the nucleus (S1B Fig). We validated EPO-induced STAT5 enhancer occupancy using two predicted target genes: *Bcl2l1* and *Abcg2* [30] (see Methods). A pSTAT5 antibody which recognises both pSTAT5a and pSTAT5b (see Methods) was best able to enrich for DNA at the *Bcl2l1* enhancer (S1C and S1D Fig). We also detected EPO-dependent pSTAT5 occupancy at one of two reported enhancers in the *Abcg2* gene, a known target of STAT5 in response to prolactin in mammary epithelium (S1E and S1F Fig) [30]. Based on these pilot studies, we undertook ChIP-seq using a pool of five biological replicates and matched input DNA samples (see Methods). A total of 302 peaks were called by MACS2 [31]; 23% of these fall within promoters (<1kb from TSS), whereas most reside within introns or intergenic regions (Fig 1A). The 50 peaks with highest enrichment are listed in Table 1 along with distance to the nearest TSS and gene feature. A full list of peaks with genome co-ordinates is available in S1 Table.

**Table 1:** Top 50 pSTAT5 ChIP-seq peaks.

Fold enrichment	Summit position (mm9)	Closest TSS symbol	Nearest Gene Name	Distance to TSS	Feature
**40.0**	chr2:154198466	*Cdk5rap1*	CDK5 Regulatory subunit associated protein 1	-11	promoter
**35.8**	chr9:107198989	*Cish*	Cytokine-inducible SH2 containing protein	31	promoter
**34.4**	chr2:167590770	*A530013C23Rik*		-74062	intergenic
**25.9**	chr5:66123040	*N4bp2*	NEDD4 binding protein 2	31720	distal
**25.8**	chr11:31744046	*Cpeb4*	Cytoplasmic Polyadenylation Element Binding Protein 4	28165	distal
**22.6**	chr2:152615403	*Cox4i2*	Cytochrome C oxidase subunit IV isoform 1	-35494	intron
**22.1**	chr6:31563009	*Podxl*	Podocalyxin-like	-49072	distal
**20.8**	chr7:132615741	*Nsmce1*	Non-SMC element 1 homolog	19315	CDS
**20.7**	chr2:152175767	*Gm14164*	Predicted gene 14164	-4362	intron
**20.3**	chr7:3581598	*Prpf31*	Pre-mRNA processing factor 31	-11	promoter
**20.0**	chr6:122713229	*Slc2a3*	Solute carrier family 2 facilitated glucose transporter member 3	-20466	distal
**18.8**	chr8:87500489	*Junb*	Jun B proto-oncogene	2158	distal
**18.1**	chr14:22626331	*Vdac2*	Voltage-dependent anion channel 2	24452	distal
**18.0**	chr7:4695193	*Suv420h2*	Suppressor of variegation 4–20 homolog 2	-3464	intron
**17.5**	chr18:32702165	*Gypc*	Glycophorin C	17523	intron
**16.1**	chr9:69860285	*Gtf2a2*	General transcription factor IIA 2	72	promoter
**15.3**	chr11:117830559	*Socs3*	Suppressor of cytokine signalling 3	121	UTR
**14.8**	chr2:167875965	*Pard6b*	Par-6 family cell polarity regulator, beta	30539	distal
**14.8**	chr18:70690275	*Poli*	Poly (ADP-ribose) polymerase 1	-300	promoter
**14.8**	chr4:114732128	*Tal1*	T-cell acute lymphocytic leukemia 1	4	promoter
**14.6**	chr17:35182005	*Msh5*	MutS homolog 5	1546	intron
**14.5**	chr8:86267080	*Cd97*	Adhesion G protein-coupled receptor E5	-1870	distal
**14.3**	chr16:15863503	*Mzt2*	Mitotic spindle organising protein 2	-88	promoter
**14.2**	chr1:133035898	*Dyrk3*	Dual-specificity tyrosine-(Y)-phosphorylation regulated kinase 3	-1087	distal
**13.9**	chr3:88936813	*Pklr*	Pyruvate kinase, liver and red blood cell	3251	distal
**13.9**	chr1:172776118	*Atf6*	Activation transcription factor 6	21784	intron
**13.8**	chr10:79580192	*Stk11*	Serine/threonine kinase 11	-911	intron
**13.8**	chr19:41520412	*Pik3ap1*	Hematopoietic cell signal transducer	-60852	intergenic
**13.8**	chr2:179983863	*Rps21*	Ribosomal protein S21	8221	distal
**13.6**	chr3:88314216	*Lmna*	Lamin A/C	-6962	distal
**13.5**	chr1:182833199	*Pycr2*	Pyrroline-5-Carboxylate Reductase Family Member 2	1206	distal
**13.2**	chr7:151972633	*Ano1*	Anoctamin 1	-48136	distal
**13.1**	chr1:157319900	*Xpr1*	Xenotropic and polytropic retrovirus receptor 1	-55326	intergenic
**13.1**	chr6:148160725	*4732416N19*		85	promoter
**12.9**	chr3:95478327	*Adamtsl4*	Thrombospondin repeat-containing protein 1	13454	distal
**12.8**	chr7:87291129	*Mir1965*	microRNA 1965	6772	distal
**12.7**	chr11:94873704	*Samd14*	Sterile alpha motif domain containing 14	-2511	intron
**12.7**	chr15:11329674	*Tars*	Threonyl-tRNA synthetase	-261	promoter
**12.4**	chr11:45619192	*F630206G17Rik*		2393	distal
**12.3**	chr3:88936393	*Pklr*	Pyruvate kinase, liver and red blood cell	3671	distal
**12.2**	chr8:113457440	*St3gal2*	ST3 beta-galactoside alpha-2,3-sialyltransferase	-13675	intron
**12.2**	chr5:105840185	*Lrrc8b*	Leucine Rich Repeat Containing 8 Family Member B	4609	distal
**12.0**	chr19:32447087	*Sgms1*	Sphingomyelin synthase 1	15857	intron
**12.0**	chr6:124633853	*Lpcat3*	Lysophosphatidylcholine Acyltransferase 3	-20731	intron
**12.0**	chr9:20673620	*A230050P20Rik*		-534	intron
**11.8**	chr12:77771803	*Sptb*	Spectrin Beta	39731	intron
**11.8**	chr15:76029159	*Plec*	Plectin	-520	promoter
**11.7**	chr17:53618017	*Rab5a*	RAS-Associated Protein RAB5A	542	promoter
**11.7**	chr1:53120923	*Mstn*	Myostatin	-2416	CDS
**11.6**	chr11:69735393	*Eif5a*	Eukaryotic Translation Initiation Factor 5A	67	promoter

We undertook *de novo* motif discovery using MEME [32] and found significant enrichment for a palindromic STAT binding site or GAS element, TTCYMRGAA, within the peak regions (Fig 1B). This is consistent with similar GAS motif enrichment in other STAT5 data sets [30,33], with EMSA studies of EPO-induced GAS element binding in erythroid cells [15,26], and with detailed *in vitro* binding data for STAT5a and STAT5b [15,26,34,35]. The majority of our pSTAT5 peaks contained a central GAS element according to CentriMO [32], confirming specificity of the ChIP (S1G Fig). We also found significant enrichment of typical GATA (WGATAA) and CACCC-box (CCC-CNC-CCN) motifs within the neighbourhood of pSTAT5 peaks (Fig 1B), but no other motifs.

### Genome co-occupancy by pSTAT5, GATA1 and KLF1

Based on these enriched PWMs, we asked whether there is co-occupancy with GATA1 and KLF1 (two critical TFs for erythroid maturation) using reported ChIP-seq data [36,37]. We found 67 of the 302 pSTAT5-occupied sites were bound by KLF1 and 147 were bound by GATA1 (Fig 1C and 1D). Interestingly, the pSTAT5 occupied sites not co-occupied by GATA1 are occupied by STAT5a and/or STAT5b in different cell types [38–41] (Fig 1D). This suggests pSTAT5 regulates a common subset of genes in most cell types in which it is expressed. Such genes include generic negative feedback regulators of cytokine signalling such as *Cish* and *Socs3* and generic effectors of cell survival such as *Bcl2l1* (S1D Fig). Thus, we suggest pSTAT5 undertakes two functions in erythroid cells; a generic function which is present in all cells where cytokine receptor signalling occurs, and an erythroid-specific function. In some cases this is likely to depend upon the activity of GATA1 and/or KLF1. This has been formally shown for STAT5-induced differentiation of human CD34+ cells into erythrocytes [42]. We hypothesised many pSTAT5-occupied regions are likely to be enhancers. For those sites which overlap KLF1 and GATA1-occupied sites this provides evidence for such a role [20,43], but we sought additional evidence from H3K4me1 and H3K4me3 ChIP-seq, and DNase1 HS in primary fetal liver cells (S2 Fig). Indeed, pSTAT5-occupied sites correspond to erythroid enhancers or promoters (S2 Fig).

### Immediate EPO-induced transcriptome changes

**Fig 2:**
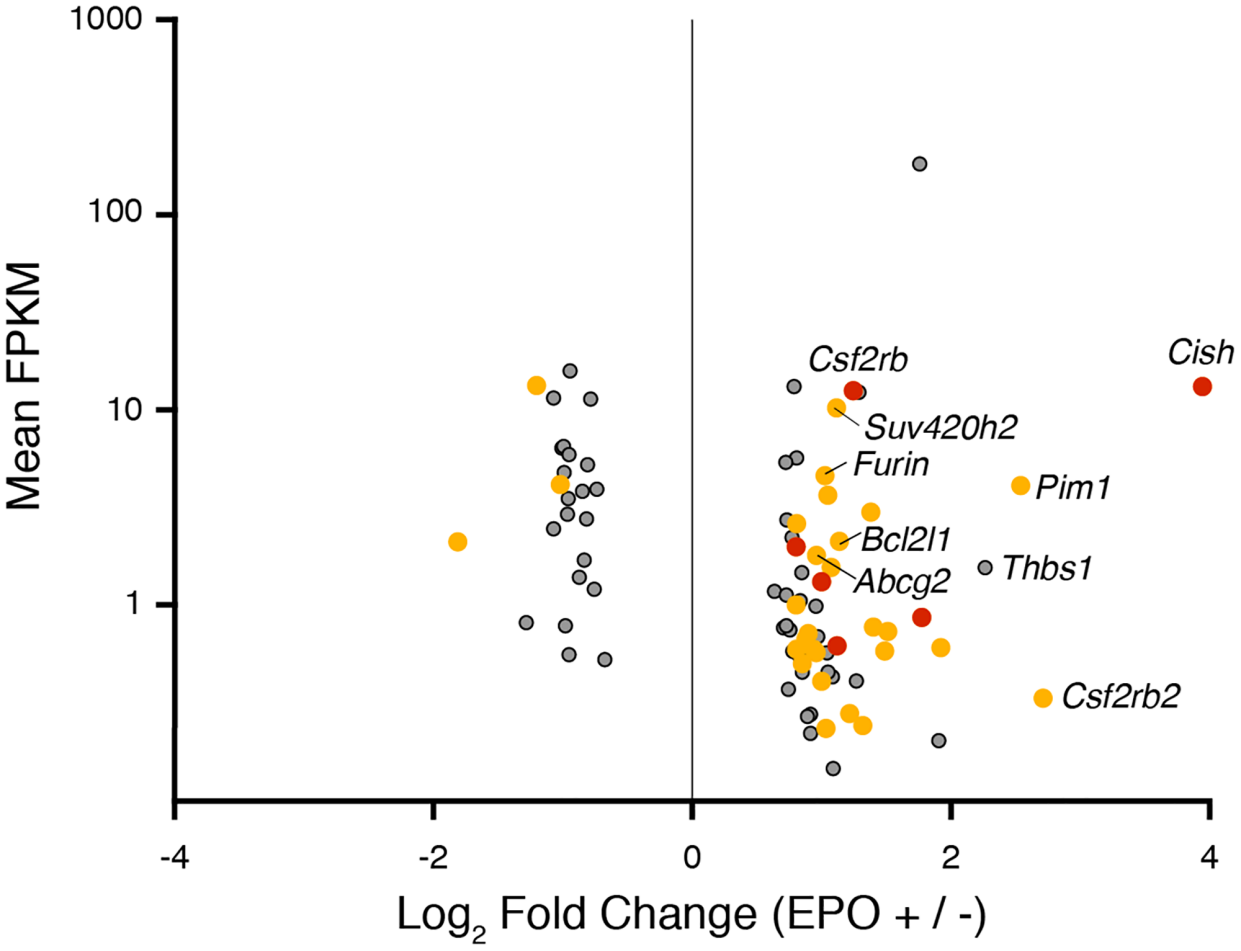
EPO-induced changes in erythroid gene expression. Statistically significant DEGs are plotted as fold change (EPO +/-) versus mean FPKM between conditions. Yellow points represent genes where pSTAT5 binds within 100 kb of TSS (presumably enhancers) and red points are DEGs with promoter bound pSTAT5. Grey points represent genes with no significant pSTAT5 binding site within 100 kb.

To determine the immediate transcriptional responses to EPO we performed 4sU-labelling of newly transcribed RNA in murine J2E cells for 30 mins following 10 mins of induction with EPO (10U/ml) or without EPO as a control (S1A Fig). Previous work demonstrated rapid upregulation of transcription of specific genes such as *Cish* within 10–30 minutes of EPO stimulation [44,45], so we focused on this early time point. We harvested total RNA, enriched for 4sU-labelled RNA and confirmed enrichment (~1000-fold) by qRT-PCR for *Hprt* and *β-globin* (S3A Fig). We found 63 genes significantly induced by EPO whereas 24 were repressed (Fig 2 and Table 2). There was a strong correlation between biological replicates (r^2^>0.95) (S3B and S3C Fig). Many of these rapidly responding genes are well known EPO-induced genes such as *Cish*, *Bcl2l1* and *Pim1* [25,26,45,46]. There is also a strong overlap between our gene list and rapidly EPO-induced genes from primary erythroid cells [47].

**Table 2:** Immediate early EPO-induced genes.

Gene ID	Gene name	Fold change	Function
***Cish***	Cytokine-inducible SH2-containing protein	8.4	JAK/STAT signalling inhibitor
***Pim1***	Pim-1 proto-oncogene	3.7	Serine threonine kinase
***Thbs1***	Thrombospondin	3.3	Mediator of cell-cell interactions
***Csf2rb2***	Colony stimulating factor 2 receptor, beta 2	2.6	βc subunit of GM-CSF and IL5 receptors
***Plek2***	Plekstrin 2	2.4	Cytoskeletal arrangement
***Nek6***	Nima-related kinase 6	2.3	Cell cycle regulation
***Vaultrc5***		2.3	
***Tshz1***	T-shirt zinc finger homeobox 1	2.2	Developmental regulation
***Csf2rb***	Colony stimulating factor 2 receptor, beta	2.1	βc subunit of GM-CSF and IL5 receptors
***Ralb***	V-Ral simian leukemia viral oncogene homolog B	2.1	GTPase, immune signalling
**5730420D15Rik**		2.0	
***Samsn1***	SAM domain SH3 domain and nuclear localization signals 1	2.0	Regulates cytoskeletal rearrangement, cell polarisation
***Bcl2l1***	B-cell lymphoma 2 like 1	2.0	Cell survival and apoptosis
***Traf5***	TNF receptor-associated factor 1	1.9	Signal transduction Anti-apoptotic
***Samd14***	Sterile alpha motif domain containing 14	1.9	Protein binding
***Hectd1***	HECT domain-containing E3 ubiquitin protein ligase	1.9	E3 ubiquitin protein ligase
***Suv420h2***	Suppressor of variegation 4–20 homologue 2	1.9	H4K20 trimethylase
**2310014L17Rik**		1.9	
***Pde4d***	Phosphodiesterase 4D, cAMP specific	1.9	Phosphodiesterase
***Rtn4***		1.9	
***Clint1***	Clathrin Interactor 1	1.9	Stimulation of clathrin assembly
***Kat2b***	Lysine acetyltransferase 2B	1.9	Transcriptional regulator
***Furin***	Furin (Paired basic amino acid cleaving enzyme)	1.8	Endoprotease
***Arhgap28***	Rho GTPase activating protein 28	1.8	GTPase activator activity
***Abcg2***	ATP-binding cassette, sub family G	1.8	Efflux transport protein
***Ripk1***	Receptor (TNFRSF)-interacting serine-threonine kinase 1	1.8	Inflammatory and cell-death signalling
***Plcl2***	Phospholipase C-like 2	1.8	Phospholipase Signal transduction
***Vrk2***	Vaccinia related kinase 2	1.8	Cell growth and survival regulation
***Ppard***		1.7	
***Tle4***	Transducin-like enhancer of split 4	1.7	G protein-coupled receptor signalling
***Zfp933***	Zinc finger protein 933	1.7	Protein binding
***Atf6***	Activating transcription factor 6	1.7	Gene activation during ER stress
***Ralgps2***	Ral GEF with PH domain and SH3-binding motif 2	1.7	Phospholipid binding
***Mroh1***	Maestro heat-like repeat family member 1	1.7	Protien coding
***Btk***	Bruton A gammaglobulinemia tyrosine kinase	1.7	B-cell development
***Exoc6***	Exocyst complex compound 6	1.7	Intercellular trafficking
***Akt3***	V-akt murine thymoma viral oncogene homolog 3	1.7	Insulin and GF signalling regulation
***Tcf4***	TPR-containing transcription factor IIIC subunit	1.7	Protein assembly
***Adk***	Adenosine kinase	1.7	Adenosine regulator
***Nt5c3***	5’-Nucleosidase, cytosolic 3	1.6	Catalyst of dephosphorylation
***Mms19***	MMS19 nucleotide excision repair homolog	1.6	Iron-sulphur protein assembly complex
***Vcl***	Vinculin	1.6	Cell-cell junctions
***Lrch1***	Leucine-rich repeats and calponin homology domain containing 1	1.6	Potential structural role
***Hells***	Helicase, lymphoid-specific	1.6	DNA repair and replication mechanisms
***Sgms1***	Sphingomyelin synthase 1	1.6	Transmembrane protein
***Epb4***.***1l5***	Erythrocyte membrane protein band 4.1 like 5	1.6	Establishment of epithelial cell polarity
***Pvt1***	Plasmacytoma variant translocation 1 oncogene	1.6	lncRNA
***2610507B11Rik***	RIKEN cDNA 2610507B11	1.6	Membrane trafficking
***Scd2***	Stearoyl-CoA desaturase 2	1.6	Fatty acid biosyntesis
***Cnot6***	CCR4-NOT homolog transcription complex subunit 6	1.6	Transcriptional regulation
***Snx9***	Sorting nexin 9	1.6	Intracellular trafficking
***Lin28b***	Lin-28 homolog B	1.6	Cardiac progenitor differentiation
***Lin52***	Lin-52 DREAM muvB core complex component	1.6	Cell cycle regulation
***BC016423***	BC016423	1.6	Protein coding
***Ptpn4***	Protein tyrosine phosphatase non-receptor type 4	1.6	Cellular growth signalling
***Slmap***	Sarcolemma associated protein	1.6	Myoblast fusion
***Rbm25***	RNA binding motif protein 25	1.6	Regulation of Bcl2l1 splicing
***Rad51b***	RAD52 paralog B	1.6	Homeostasis and homologous recombination
***Kansl1***	KAT8 regulatory NSL complex subunit 1	1.6	Histone acetylation
***Vmp1***	Vacuole membrane protein 1	1.5	Cytoplasmic vacuolization
***Tnrc6b***	Trinucleotide repeat containing 6B	1.5	PI3 kinase signalling
***Rabgap1l***	RAB GTPase activation protein 1-like	1.5	Protein coding

There was an overlap between rapidly induced transcripts and nearby ChIP-seq peaks but this was not always the case (Fig 2 and S2 Table). In fact, we found three scenarios: (i) EPO-induced genes with a nearby pSTAT5 peak (likely direct targets), (ii) EPO-induced genes with no nearby peak (likely STAT5-independent), and (iii) pSTAT5 peaks without any change in expression of the nearest gene (S1 Fig). For the second scenario, some DEGs may be dependent upon alternative EpoR-generated signals, such as PI3K-AKT or MAPK-ERK pathway signals, which induce gene expression changes via alternate transcription factors to STAT5. For the third scenario, a putative pSTAT5-regulated gene may not be the closest gene to the peak, but reside hundreds of kilobases away. Thus, we could miss-assign some true immediate target genes regulated by distant enhancers. This possibility is difficult to resolve without chromatin conformation capture data. Alternatively, there may have been a delay between pSTAT5 binding and induction of gene expression in some cases. Indeed, there is a dynamic transcriptional response to EPO stimulation [45,47]. To try to resolve these alternate possibilities, we integrated published dynamic CAGE data from J2E cells and also performed qRT-PCR at multiple time points up to 24 hours post EPO stimulation (S1 Fig).

### Direct pSTAT5 target genes with immediate early induction

**Fig 3:**
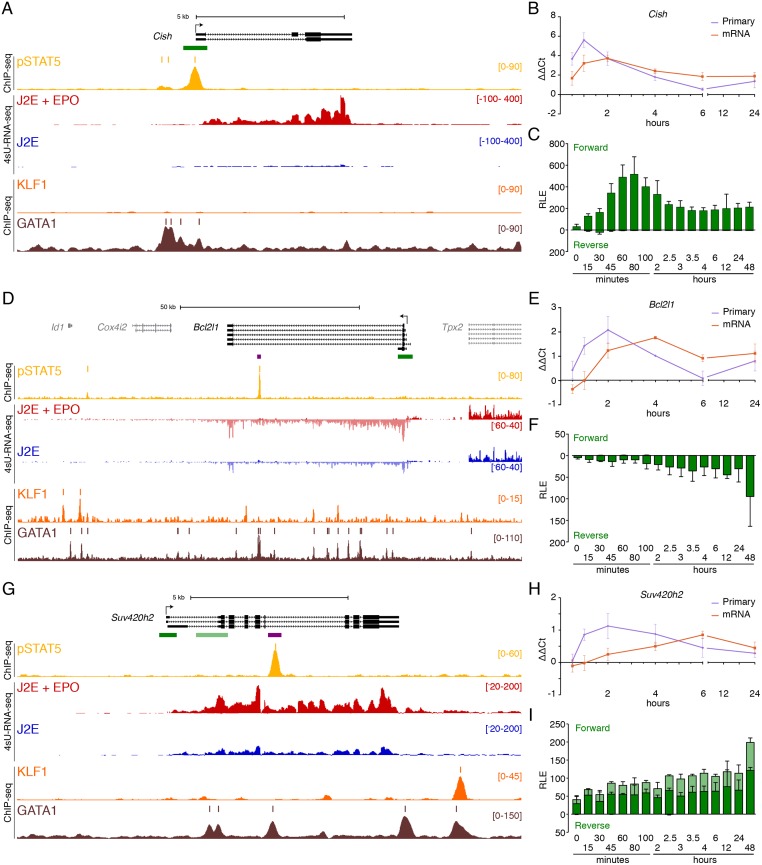
Immediate direct transcriptional targets of EPOR-pSTAT5 signalling. (A) ChIP-seq and 4sU-RNA-seq in erythroid cells following 30 mins of EPO induction across the *Cish* gene. Read density per million mapped reads (y-axis) for pSTAT5 (ochre), KLF1 (orange) and GATA1 (brown) ChIP. Coloured bars above each track represent peak summits as called by MACS2. Read density profiles (y-axis) for 4sU-RNA-seq from J2E cells (blue) and J2E cells post EPO-induction (red) are displayed for both forward (+ values) and reverse (- values) strands. A schematic of gene structure and alternative transcripts from RefSeq is shown in black with scale bar. The green bar represents the region selected for CAGE tag counts shown in (C). (B) qRT-PCR for *Cish* primary pre-spliced transcripts and processed mRNA over a 24-hour time course following EPO stimulation. (C) CAGE tag counts expressed as relative log expression (RLE) over the indicated pSTAT5-occupied promoter (green bar in panel A) from 0 to 24 hours post EPO stimulation. ChIP-seq and 4sU-RNA-seq across the *Bcl2l1* (D) and *Suv420h2* (G) genes. Overall design and colour coding of tracks is the same as for (A). Purple bars indicate pSTAT5-occupied intronic enhancers. Dynamic EPO-induced CAGE tags over this region are shown in S4 Fig. qRT-PCR for *Bcl2l1* (E) and *Suv420h2* (H) primary pre-spliced transcripts and processed mRNA over a 24-hour time course following EPO stimulation as in (B). CAGE tag counts for *Bcl2l1* (E) and *Suv420h2* (I) over promoters (dark green) and alternative promoter (light green) from 0 to 24 hours post EPO stimulation as in (C).

For many DEGs with a proximal ChIP-seq peak, there was rapid induction of primary transcription (within 30–60 mins) which was processed leading to a slower accumulation of mRNA. For example, *Cish* has a robust pSTAT5 ChIP-seq peak at its promoter (Fig 3A, yellow track) and weaker peaks about 500bp upstream which overlap with GATA1 occupied regions (brown track). There are four typical palindromic GAS elements near the centre of the peak which respond to EPO in luciferase assays [26], and bind pSTAT5 by EMSA. There is induction of newly transcribed RNA over the gene body (red track) compared with unstimulated cells (blue). There is also a 4-fold upregulation of *Cish* primary transcripts by qRT-PCR at 30 mins, and later upregulation of mRNA and promoter CAGE tag counts at 60 mins (Fig 3B and 3C). Thus, these complimentary data sets provide a detailed and consistent account of the direct transcriptional response of *Cish* to pSTAT5.

Similarly, the *Bcl2l1* gene contains a strong pSTAT5 ChIP-seq peak towards the 3′ end of a long second intron; this site overlaps with two GATA1 ChIP-seq peaks (purple bar, Fig 3D). Some GATA1 peaks overlap with KLF1 peaks (orange) but the pSTAT5 peak does not. The primary transcripts for *Bcl2l1* are slightly increased at 30 mins but are not maximally induced until ~2 hours post EPO stimulation (Fig 3E). Processed mRNA induction peaks at ~4 hours post-stimulation which corresponds to the timing of maximal accumulation of promoter and enhancer CAGE tags (Fig 3F and S4A Fig). So, *Bcl2l1* is a direct target of EPO-pSTAT5 but the dynamics of mRNA accumulation are delayed compared to *Cish*.

### Direct pSTAT5 target genes with delayed RNA induction dynamics

**Fig 4:**
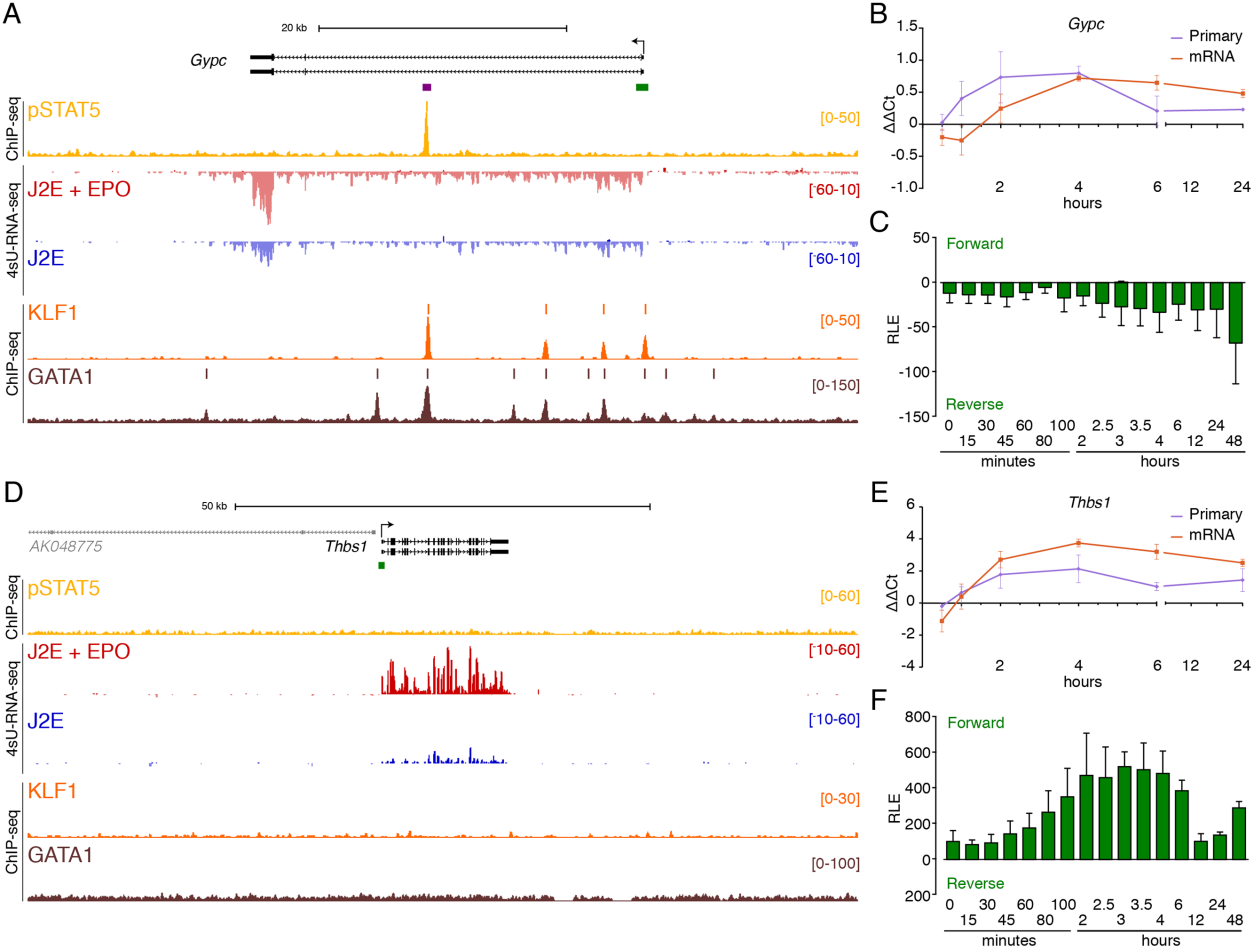
Delayed pSTAT5-dependent and independent EPO-induced gene expression. ChIP-seq and 4sU-RNA-seq in erythroid cells following 30 mins of EPO induction across the *Gypc* (A) and *Thbs1* (D) genes. The overall design and colour coding of tracks is the same as for Fig 3. qRT-PCR and CAGE tag counts as in Fig 3B and 3C for *Gypc* and Fig 3E and 3F for *Thbs1* respectively. Bidirectional CAGE tags over the intronic enhancer (purple bar in panel A) are shown in S4C Fig. *Gypc* provides an example of delayed transcriptional activation by pSTAT5, whereas *Thbs1* appears to be activated independently of pSTAT5.

The *Suv420h2* gene has a strong intronic pSTAT5 ChIP-seq peak and obvious 4sU RNA induction at 30 mins (Fig 3G, red versus blue tracks), but primary transcripts peak at 2 hours and remain high at 4 hours post EPO treatment (Fig 3H). Similarly, CAGE tags at the promoter and first intron both slowly accumulate in response to EPO (Fig 3I). Interestingly, CAGE tags accumulate more rapidly at the pSTAT5-occupied enhancer in intron 6 (purple bar in Fig 3I and S4B Fig). There was significant upregulation of 4sU-RNA for *Suv420h2* at 30 mins but induction was greater at later time points. We hypothesised additional direct pSTAT5 target genes with similarly delayed induction could be missed in the 30 min 4sU RNA sample. So, we undertook qRT-PCR and CAGE data analysis at multiple time points for 24 hours post EPO stimulation for candidate delayed target genes (Fig 4).

*Gypc* encodes Glycophorin C, a known direct target of KLF1[22,48]. It has a large first intron with a robust central pSTAT5 peak (Table 1). There are multiple evenly spaced KLF1 and GATA1 peaks throughout the 5′ part of intron 1 with the genomic architecture of a super enhancer (Fig 4A) [49,50]. The strongest KLF1/GATA1 co-occupied region is also bound by pSTAT5 whereas the lesser peaks and the promoter are not. There is some baseline expression of *Gpyc* in the absence of EPO (blue track) but limited induction of 4sU-labelled RNA by 30 mins (red), so *Gpyc* was not called as a DEG. However, qRT-PCR and promoter CAGE data clearly show significant induction by EPO (Fig 4B and 4C), albeit delayed (>30 mins). We found similar delayed induction of *Podxl* which has two strong upstream pSTAT5 bound regions (Table 1 and S5A Fig, ochre track), both of which are co-occupied by GATA1 (brown) and KLF1 (orange). Once again there is delayed induction of primary RNA, mRNA and CAGE tags (S5B and S5C Fig).

Lastly, there are some genes which have a robust pSTAT5 ChIP-seq peak nearby but display no obvious induction even at late time points. For example, there is a strong pSTAT5 ChIP-seq peak at the *Cdk5rap1* promoter (Table 1 and S5D Fig), but no evidence or induction of transcription by 4sU-RNA-seq, qRT-PCR or promoter CAGE data (S5E and S5F Fig). It is possible the promoter-bound pSTAT5 site is actually a distant enhancer for some other DEG, but this is not obvious upon inspection of the UCSC Browser. So, there appear to be some pSTAT5 bound sites which do not direct local gene expression. The function of pSTAT5 at these sites is uncertain.

### EPO induced pSTAT5-independent genes

We also considered genes which showed rapid induction in response to EPO (called as DEGs), but which had no pSTAT5 ChIP-seq peak within 50kb of the TSS. For example *Thbs*, which encodes thrombospondin, was induced 3.3 fold (Table 2) with obvious accumulation of 4sU RNA (Fig 4D, red). We confirmed gene induction by qRT-PCR and CAGE with similar dynamics to direct pSTAT5 target genes (Fig 4E and 4F). However, there is no pSTAT5 peak within 50 kb of the *Thbs1* TSS (Fig 4D). The data for *Furin* is similar although there is a weak pSTAT5 peak downstream of the neighbouring gene, *Fes*, which could be a distant 3′ enhancer for *Furin* (S5G–S5I Fig). Without 3C data this possibility remains speculative. In short, we found 38 EPO-induced genes which are not obvious direct pSTAT5 target genes (Fig 2 and S1A Fig). One likely scenario is they are responding to a pSTAT5-independent signal, such as a PI3K or MAPK.

## Discussion

### New EPO-responsive genes

We employed ChIP-seq to determine pSTAT5 occupancy and 4sU-RNA-seq to detect immediate transcriptional targets of EpoR signalling in murine erythroid cells. We found >300 pSTAT5-occupied regions in the erythroid genome. A reduction in the q value threshold from 0.01 to 0.2 only increased peak calls by about 25%, but it also reduced the quality of the peaks (i.e. central GAS sites fell to 50%), so the true number of pSTAT5-occupied sites is low compared with other erythroid TFs. It is important to remember we undertook the ChIP at one time point after EPO stimulation and in cell lines immortalised at the early erythroblast stage of differentiation. Additional pSTAT5 occupancy may occur at delayed times after EPO stimulation or in more differentiated cells. About 15% of the peaks occur at gene promoters but the majority occur at intronic or intergenic sites. These mostly have features characteristic of enhancers such as bi-directional CAGE tags, the H3K4me1 mark and DNase 1 hypersensitivity.

Of the 63 genes identified as EPO immediate early induced, some were unexpected. Manual curation of the encoded proteins places most of them within four functional categories (Table 2) including proteins involved in: (i) receptor down regulation or degradation, (ii) signalling or proliferation responses, (iii) pro-survival pathways, and (iv) nuclear functions such as transcription, chromatin modification and RNA splicing. While 38 did not have obvious pSTAT5 ChIP-seq peaks at promoters or intronic regions, there may be more distant pSTAT5-occupied enhancers. However, it is likely that many will be pSTAT5-independent EPO target genes. Small molecule inhibitors of STAT5-independent pathways and ChIP-seq for other transcriptional effectors of EPO signalling such as FOXO3 [51] might help to resolve this issue.

### STAT5-dependent negative feedback loops down regulate the EpoR

We found some genes with known roles in down regulation of EpoR signalling are direct targets of pSTAT5; e.g. *Cish* and *Socs3* (S6 Fig). CISH is recruited to activated pY401 in the EpoR [26]. Like many SOCS family members, CISH recruits a complex of proteins including elongin B, elongin C, cullin5 and Rbx2 which together provide E3 ubiquitin ligase activity to add ubiquitin to nearby substrates [52] leading to degradation in the proteasome. In this way CISH provides a negative feedback to dampen EPO-induced signalling (S6 Fig). SOCS3 functions in a similar way but it also contains an N-terminal KIR peptide which has been shown to directly influence STAT5 binding by JAK2 [53] (S6 Fig). The structure of the KIR-JAK2 complex has been solved in the context of the gp130 receptor and is presumably similar for the EpoR. Interestingly, the dynamics of upregulation of CISH and SOCS3 are slightly different [45], so they can provide distinct feedback dynamics.

**Fig 5:**
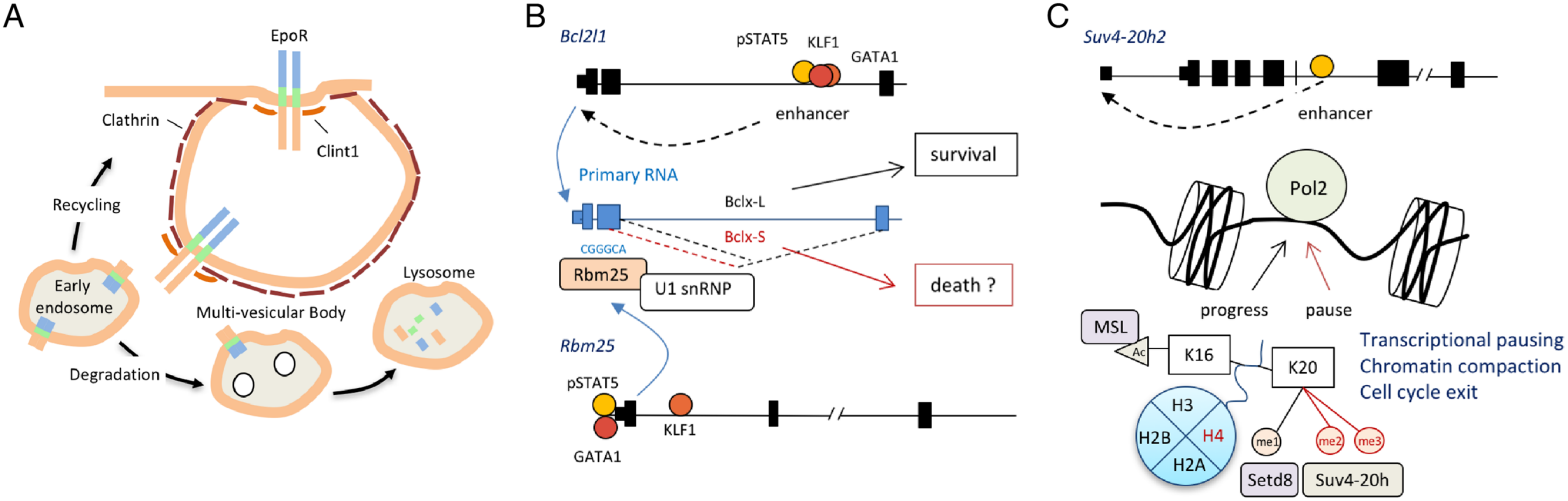
Erythroid genes and biological pathways directly regulated by pSTAT5. (A) *Clint1*, also known as *EpsinR*, is a direct target gene of pSTAT5 (see Table 1). Clint1 directly interacts with Clathrin to facilitate clathrin-mediated endocytosis (CME) of the EpoR and associated proteins. The EpoR can either be recycled from early endosomes to the cell surface for re-use, or degraded via late endosomes, multi-vesicular bodies and eventually lysosomes. (B) The *Bcl2l1* gene encodes Bcl-x which is essential for terminal erythroid differentiation. pSTAT5 binds with KLF1 and GATA1 to upregulate its expression in response to EPO. pSTAT5 also binds the *Rmb25* promoter with GATA1 to drive expression. The encoded protein, RBM25, is an RNA-binding protein which can bind into the second exon of the *Bcl2l1* RNA via a CGGGCA element to induce preferential splicing to generate Bcl-x(S) isoform in preference to the Bcl-x(L) isoform. The former isoform is pro-apoptotic in some contexts but may play an independent role in erythroid maturation and enucleation. (C) The *Suv420h2* gene is a direct target of pSTAT5 via an intronic enhancer. SUV4-20h2 is a histone methyltransferase which recognises H4K20me1 and adds two additional methyl groups. H4K20 is first methylated by Setd8/PR-Set7, a methyltransferase that is essential for erythropoiesis. H4K20me3 inhibits access to H4K16 by the MSL acetylase machinery. Acetylation at H4K16 is associated with active gene transcription whereas H4K20me2/3 is associated with transcriptional pausing. So, upregulation of *Suv420h2* by EPO-pSTAT5 is likely to promote global pausing of erythroid gene transcription and reduced production of RNA, a feature of terminal erythroid cell maturation. H4K20me3 has also been associated with cell cycle arrest and chromatin compaction, two hallmarks of erythroid cell maturation.

Clint1 (EpsinR) is involved in clathrin-mediated endocytosis (CME) [54], which is the mechanism by which erythroid progenitors procure iron via transferrin-iron binding to the transferrin receptor-1 (*Tfr1*); it has also been reported to be involved in EpoR internalisation [55]. The N-terminal ENTH domain of Clint1 binds cargos and the central domain binds clathrin and the AP-1 complex which mediates CME. So, we suggest EpoR downregulation at the cell surface may be facilitated by Clint1 which is likely to associate with clathrin-coated vesicles to enhance internalisation (Fig 5A).

### EPO-induced changes in RNA splicing factor, RBM25

The *Rbm25* gene is induced by EPO via binding of pSTAT5 to an intronic enhancer (Fig 5B). It encodes an RNA-binding protein which has been reported to be involved in alternative splicing. RBM25 binds the CGGGCA sequence in exon 2 of *Bcl2l1* RNA to stabilize pre-mRNA-U1 snRNP binding, which favours generation of the Bcl-x(S)-coding in preference to the Bcl-x(L)-coding mRNA [56]. So, EPO could enhance red blood cell survival via regulation of *Rbm25* and thereby splicing of *Bcl2l1* (Fig 5B).

### Nuclear compaction and H4K20 methylation

The erythroid nucleus must undergo compaction prior to enucleation. Setd8 (PR-Set7) is a lysine methyltransferase which induces mono-methylation at histone H4K20. It is essential for nuclear compaction and enucleation [57]. We found *Suv420h2* is a direct target of pSTAT5 via a putative intronic enhancer (Figs 3G and 5C). Suv420h2 is a methyl transferase which adds methyl groups to mono-methylated K20 of histone H4, so its function is dependent upon the prior activity of Setd8. The biological function of the di- and tri-methyl marks on H4K20 is controversial. It has been reported to play roles in transcriptional pausing [58], chromatin condensation, and cell cycle control [59]. All of these activities are critical for terminal erythroid maturation. So, we postulate H4K20 tri-methylation by Suv420h2 is essential for terminal erythroid differentiation (Fig 5C).

## Materials and methods

### Cell culture and treatment with erythropoietin

The J2E cell line [60] was maintained in 10% FBS, 1% GlutaMAX^™^ (ThermoFisher; #35050061), and 1% Penicillin-Streptomycin (ThermoFisher; #10378016). Cells were treated with erythropoietin (EPO) at 10 U/mL prior to harvesting for RNA and ChIP [45] (S1A Fig).

### qPCR primer design

ChIP primers were designed to determine occupancy at predicted binding sites according to STAT5 ChIP performed in mammary tissue [30] (S3 Table). Primers for analysing 4sU-RNA enrichment and DEG validation (S4 Table) were designed to distinguish primary transcripts covering intronic and exonic regions, and mature RNA transcripts incorporating splice junctions.

### 4-Thiouridine labelling and RNA-seq

4-Thiouridine (4sU)-labelling of newly transcribed RNA with 500 μM 4sU (Sigma; #T4509), subsequent isolation, enrichment, and RNA sequencing were performed as recently described [37].

### Western blotting

Western blots were incubated with anti-phosphoSTAT5 (1:2000) in 1% BSA overnight. pSTAT5 protein was detected by chemiluminescence using the Pierce ECL Kit (Life Technologies; #32209) as per the manufacturer’s instructions following incubation with anti-rabbit IgG-HRP (Cell Signalling Technology; #7074S) (1:10,000) in 1% BSA.

### Chromatin immunoprecipitation and ChIP-seq

ChIP was performed as previously described [37]. We tested antibodies to Stat5a (L-20) (Santa Cruz; #sc-1081) as reported in [41], and anti-phospho-STAT5 (Life Technologies; #716900). Optimisation of chromatin fragmentation by sonication and antigen retention were performed by gel electrophoresis and Western blotting, as per ENCODE recommendations [61].

### Bioinformatics

Computational analysis of raw sequencing data was performed as previously described [37]. Heatmaps of signal intensity were generated using EaSeq [62] and regions were sorted according to hierarchical clustering using the nearest neighbour chain algorithm. Clustering was based on values from all datasets quantified from 200 bp surrounding the pSTAT5 peak summits. The area was divided in to 50 bins and values were log transformed and normalized to the maximum signal in the clustered areas.

### Accession numbers

All sequencing data generated by this study have been deposited in the Gene Expression Omnibus (GEO) under the accession GSE94301. Supporting DNase I data were from GSE37074, ChIP-seq datasets were accessed from GSE31039 (H3K4me1 and H3K4me3), GSE51338 (GATA1), and additional STAT5 ChIP-seq were from GSE36890, GSE34986, GSE31578 and GSE40930.

## Supporting information

S1 Fig (A) Overview of experimental design and results. The J2E murine erythroid cell line was stimulated with EPO (10 U/ml) for the indicated times. An analog of uracil, 4-thiouridine (4sU), was added after 10 minutes to label newly transcribed RNA. 4sU-labelled RNA was isolated after 30 minutes of labelling (see Methods) and used to generate sequencing libraries. DNA was cross-linked at 30 minutes post-EPO stimulation for pSTAT5 ChIP-seq. qRT-PCR samples were collected at indicated time points and CAGE libraries were generated by the FANTOM5 consortium as reported [28]. (B) Western blot for pSTAT5 in J2E cells pre- and 30 mins post-stimulation with EPO (10 U/ml). Cytoplasmic and nuclear extracts were loaded at ‘cell equivalent’ volumes. (C) EPO-induced pSTAT5 occupancy of a reported GAS element within the *Bcl2l1* gene, from mammary epithelia. ChIP was performed on 5 replicate samples following 30 mins EPO induction with the following treatments serving as controls: pSTAT5 Ab (+) or IgG control (−), and treatment with EPO (+) or without (−) for 30 mins. Enrichment of bound DNA was determined by qPCR and expressed as a % of input DNA. (D) ChIP-seq in multiple cell types following 30 mins of EPO induction across the *Bcl2l1* gene. Read density profiles for STAT5 across multiple non-erythroid tissues illustrates the basis for ChIP primer design (red tracks). Primers were designed to the previously reported GAS element, and to 1 kb upstream and downstream based upon the binding profiles. Enrichment of pSTAT5 observed in sequencing data (yellow) is consistent with qPCR (C) and prior studies. (E) EPO-induced pSTAT5 occupancy of the *Abcg2* gene enhancer. ChIP qPCR was performed as in panel (C), however enrichment was only observed at GAS element 4. (F) Read density profiles of STAT5 ChIP at the *Abcg2* gene as described in panel (D). Primers were designed to the previously reported GAS3 and GAS4 elements, and to 1 kb upstream and downstream based upon the binding profiles. Enrichment of pSTAT5 is observed only at GAS4 in sequencing data (yellow), and at a downstream erythroid specific site. (G) CentriMO analysis of discovered MEME motifs i-iii (see Fig 1) within pSTAT5-occupied regions +/- 500 bp from the peak summits. The STAT5 motif (i) is significantly enriched around the summit highlighting the specificity and quality of the ChIP. Central enrichment is also observed to a lesser extent for the GATA (ii) and KLF (iii) motifs.
(TIF)

S2 Fig (A) Density heat-map of ChIP signal centred on pSTAT5 peaks from J2E cells. The Y-axis represents individual peak regions, and the X-axis represents 10 kb surrounding the summit. Read intensities were normalized to the total number of reads across datasets and hierarchically clustered according to intensity within 500 bp of peak centre. Comparative normalized signals from primary erythroid cells (mouse fetal liver), are shown for DNase I (B), H3K4me3 ChIP (C), and H3K4me1 ChIP (D).
(TIF)

S3 Fig (A) Comparison of total RNA and isolated 4sU-labeled RNA shows selective enrichment of primary transcripts captured in the 4sU-labeled fraction. Relative levels of *Hbb-b1* and *Hprt* primary transcript by qRT-PCR, using primers targeted to both intron and exons, provide a measure of newly transcribed RNA content. Efficiency of 4sU-labeled RNA isolation from total RNA can be seen by the loss of primary transcript in the eluted fraction (total minus 4sU-labeled fraction). (B) Biological replicates of 4sU-RNA-seq libraries show very high Spearman’s correlation of gene expression r = 0.96 for EPO induced and r = 0.95 for non-induced replicates (C).
(TIF)

S4 Fig Bi-directional CAGE tag counts—A hallmark of enhancers—For the pSTAT5 bound enhancers of *Bcl2l1* (A; see Fig 3D), *Suv420h2* (B; see Fig 3G) and *Gypc* (C; see Fig 4A). (TIF)

S5 Fig Genes which are bound by pSTAT5 but display delayed or minimal changes in expression. (A) ChIP-seq, 4sU-RNA-seq, qRT-PCR and CAGE tags from erythroid cells following 30 mins of EPO induction across the *Podxl* (A-C), *Cdk5rap1* (D-F), and *Furin* (G-I) genes respectively. The overall design and colour coding of tracks is the same as for Fig 3. There are two strong pSTAT5 peaks 25kb and 50kb upstream of the *Podxl* TSS which overlap with GATA1 and KLF1 ChIP-seq peaks. However, *Podxl* is not upregulated by 4sU-RNA-seq (red track) after 30 mins, but shows weak and delayed upregulation of expression which is not significant until 4 hours in qRT-PCR and CAGE. There is a strong pSTAT5 peak at the *Cdk5rap1* promoter but no significant upregulation in 4sU-RNA-seq (red track), qRT-PCR or CAGE following EPO induction. Significant upregulation of *Furin* transcription *(*red track*)* can be seen following EPO stimulation. Only a weak pSTAT5 peak can be seen downstream of *Furin* near to the neighbouring gene, *Fes*, but there are no other pSTAT5 peaks at the *Furin* promoter or within 30 kb of the *Furin* TSS. qRT-PCR for *Furin* primary pre-spliced transcripts shows dynamic upregulation peaking at 2 hours post EPO stimulation but processed mRNA is not substantially upregulated. CAGE tags at the two alternative *Furin* promoters show basal expression and delayed gradual upregulation from the second promoter (light green bar) until 4 hours post -EPO stimulation.
(TIF)

S6 Fig The *Cish* and *Socs3* genes are both direct targets of pSTAT5 and are both rapidly upregulated by EPO. CISH binds activated pY401 in the EpoR via its SH2 domain. It recruits Elongin B via a C-terminal SOCS box and indirectly recruits Elongin C, which then recruits cullin-5 and Rbx1. This complex functions as an E3 ubiquitin ligase, to target EpoR and associated proteins for degradation in the proteasome. SOCS3 functions in a similar way via binding to pY401 but in addition it directly inhibits the kinase activity via competitive displacement of STAT5 binding in the active pocket of JAK2. It achieves this additional function via the N-terminal KIR domain which is not present in CISH.
(TIF)

S1 Table pSTAT5 ChIP-seq peaks in erythroid cells. (XLSX)

S2 Table Differentially expressed genes in 4sU-RNA-seq. (XLSX)

S3 Table qPCR primers for ChIP validation. (DOCX)

S4 Table qRT-PCR primers for gene expression validation. (DOCX)
