# Supplementary figures and images for: Direct targets of pSTAT5 signalling in erythropoiesis
Source: PLoS One. 2017 Jul 21;12(7):e0180922. doi: 10.1371/journal.pone.0180922 (PMC5521770; doi:10.1371/journal.pone.0180922)

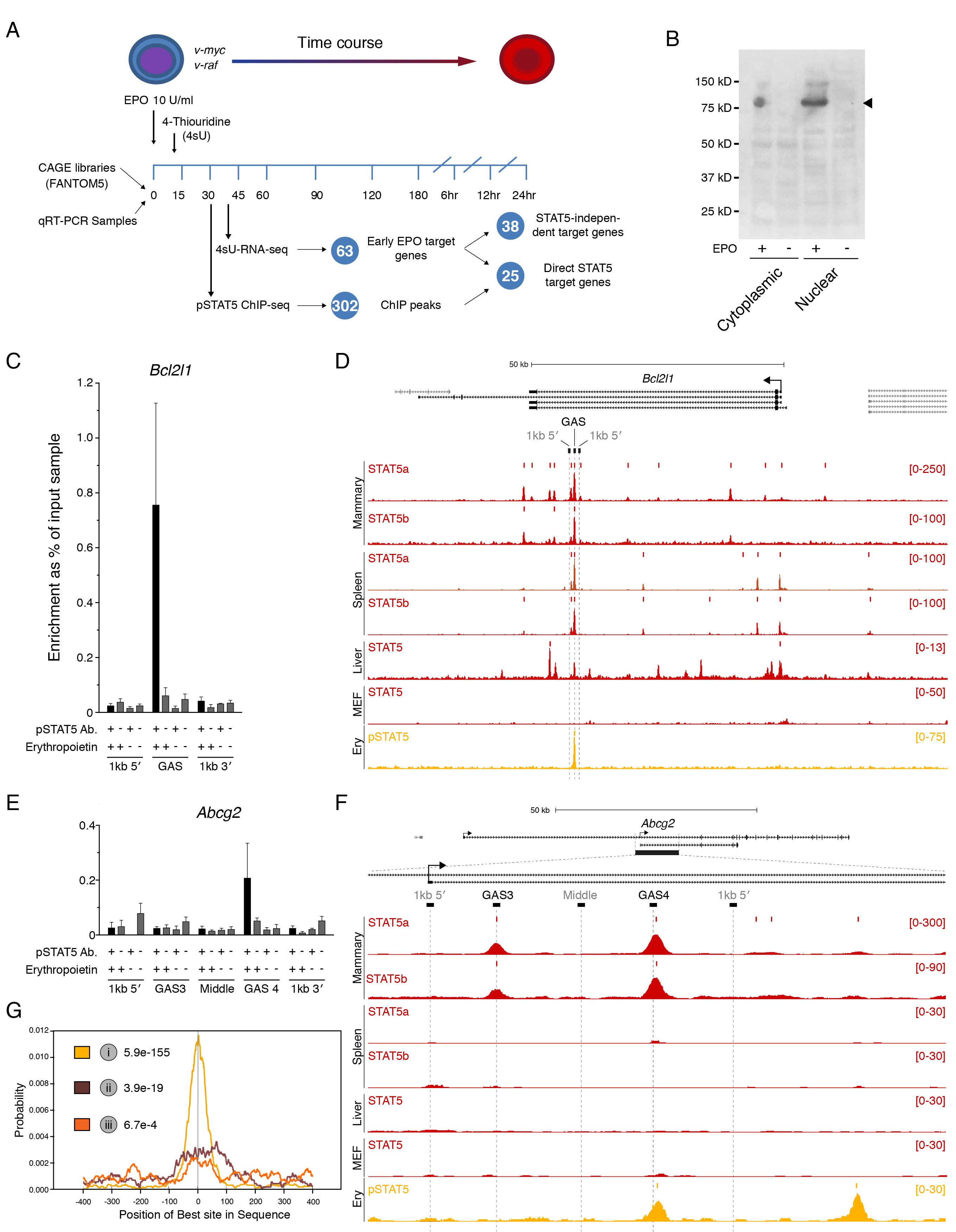

Supplement: S1 Fig — (A) Overview of experimental design and results. The J2E murine erythroid cell line was stimulated with EPO (10 U/ml) for the indicated times. An analog of uracil, 4-thiouridine (4sU), was added after 10 minutes to label newly transcribed RNA. 4sU-labelled RNA was isolated after 30 minutes of labelling (see Methods) and used to generate sequencing libraries. DNA was cross-linked at 30 minutes post-EPO stimulation for pSTAT5 ChIP-seq. qRT-PCR samples were collected at indicated time points and CAGE libraries were generated by the FANTOM5 consortium as reported [28]. (B) Western blot for pSTAT5 in J2E cells pre- and 30 mins post-stimulation with EPO (10 U/ml). Cytoplasmic and nuclear extracts were loaded at ‘cell equivalent’ volumes. (C) EPO-induced pSTAT5 occupancy of a reported GAS element within the Bcl2l1 gene, from mammary epithelia. ChIP was performed on 5 replicate samples following 30 mins EPO induction with the following treatments serving as controls: pSTAT5 Ab (+) or IgG control (−), and treatment with EPO (+) or without (−) for 30 mins. Enrichment of bound DNA was determined by qPCR and expressed as a % of input DNA. (D) ChIP-seq in multiple cell types following 30 mins of EPO induction across the Bcl2l1 gene. Read density profiles for STAT5 across multiple non-erythroid tissues illustrates the basis for ChIP primer design (red tracks). Primers were designed to the previously reported GAS element, and to 1 kb upstream and downstream based upon the binding profiles. Enrichment of pSTAT5 observed in sequencing data (yellow) is consistent with qPCR (C) and prior studies. (E) EPO-induced pSTAT5 occupancy of the Abcg2 gene enhancer. ChIP qPCR was performed as in panel (C), however enrichment was only observed at GAS element 4. (F) Read density profiles of STAT5 ChIP at the Abcg2 gene as described in panel (D). Primers were designed to the previously reported GAS3 and GAS4 elements, and to 1 kb upstream and downstream based upon the binding profiles. Enrichment [file pone.0180922.s001.tif]

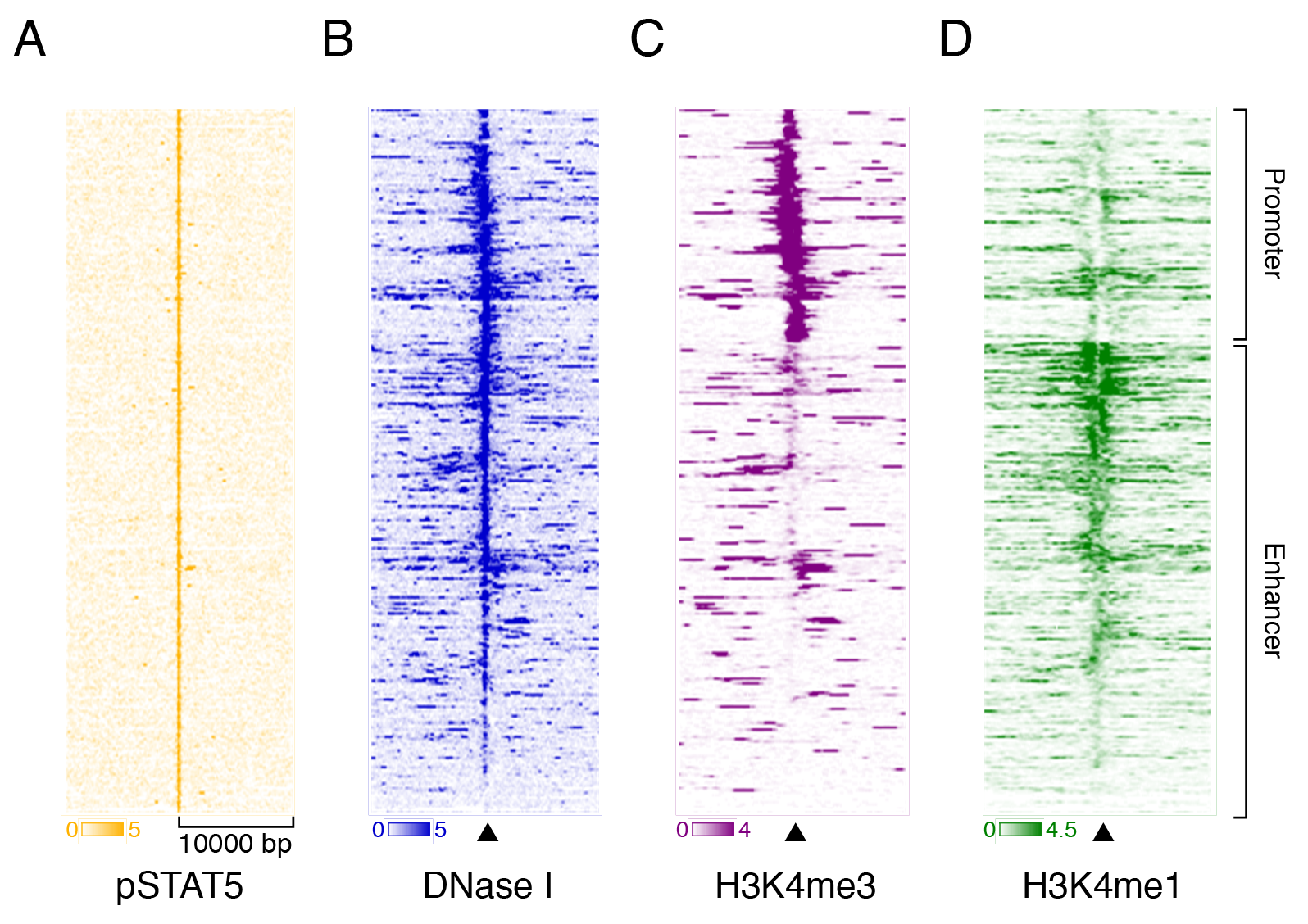

Supplement: S2 Fig — (A) Density heat-map of ChIP signal centred on pSTAT5 peaks from J2E cells. The Y-axis represents individual peak regions, and the X-axis represents 10 kb surrounding the summit. Read intensities were normalized to the total number of reads across datasets and hierarchically clustered according to intensity within 500 bp of peak centre. Comparative normalized signals from primary erythroid cells (mouse fetal liver), are shown for DNase I (B), H3K4me3 ChIP (C), and H3K4me1 ChIP (D). (TIF) [file pone.0180922.s002.tif]

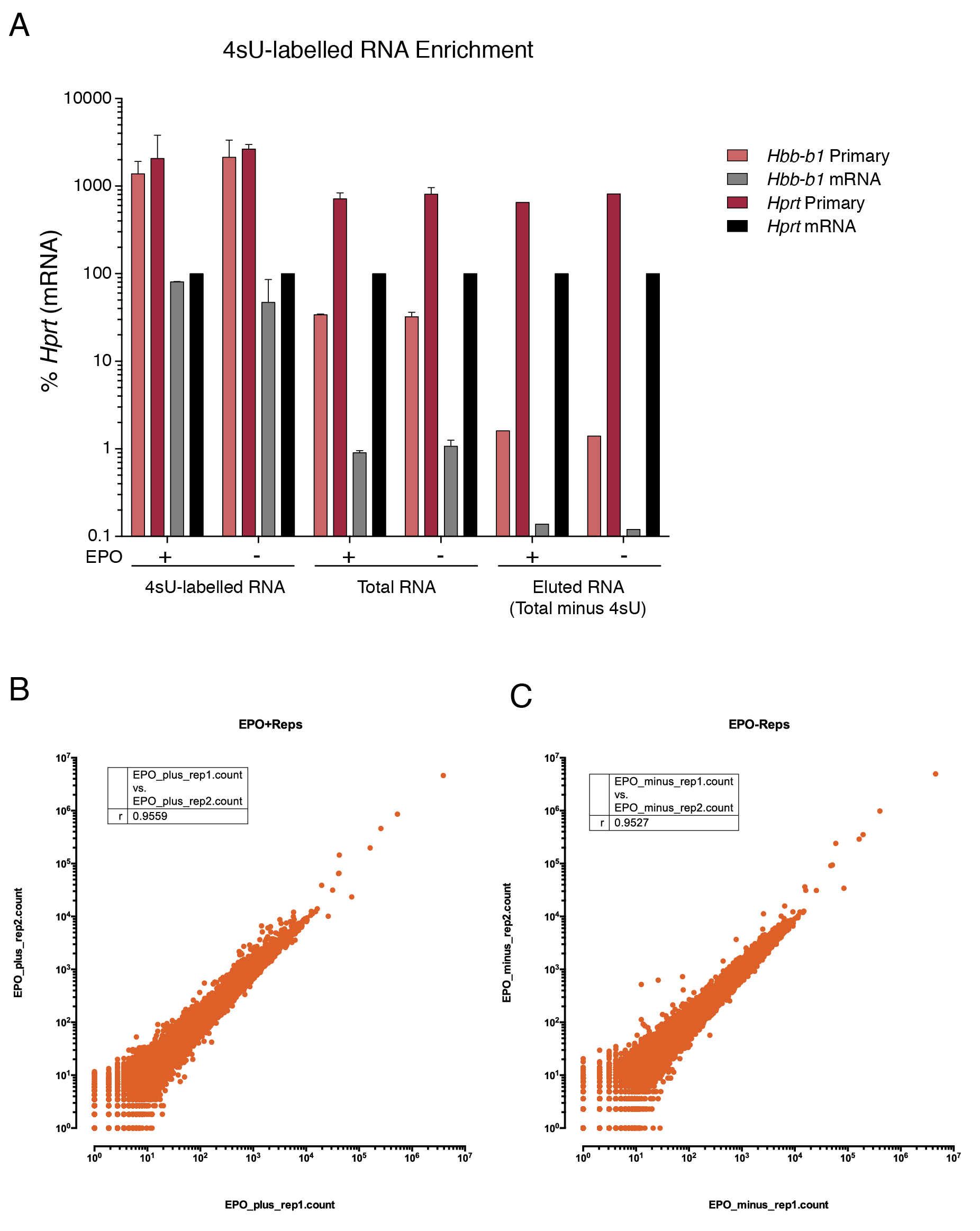

Supplement: S3 Fig — (A) Comparison of total RNA and isolated 4sU-labeled RNA shows selective enrichment of primary transcripts captured in the 4sU-labeled fraction. Relative levels of Hbb-b1 and Hprt primary transcript by qRT-PCR, using primers targeted to both intron and exons, provide a measure of newly transcribed RNA content. Efficiency of 4sU-labeled RNA isolation from total RNA can be seen by the loss of primary transcript in the eluted fraction (total minus 4sU-labeled fraction). (B) Biological replicates of 4sU-RNA-seq libraries show very high Spearman’s correlation of gene expression r = 0.96 for EPO induced and r = 0.95 for non-induced replicates (C). (TIF) [file pone.0180922.s003.tif]

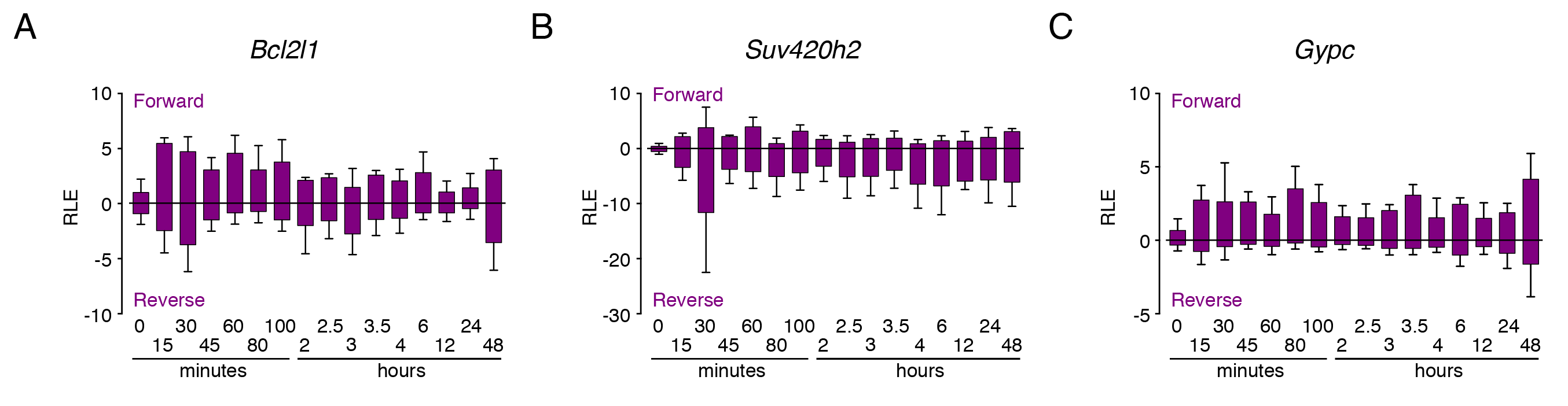

Supplement: S4 Fig — (TIF) [file pone.0180922.s004.tif]

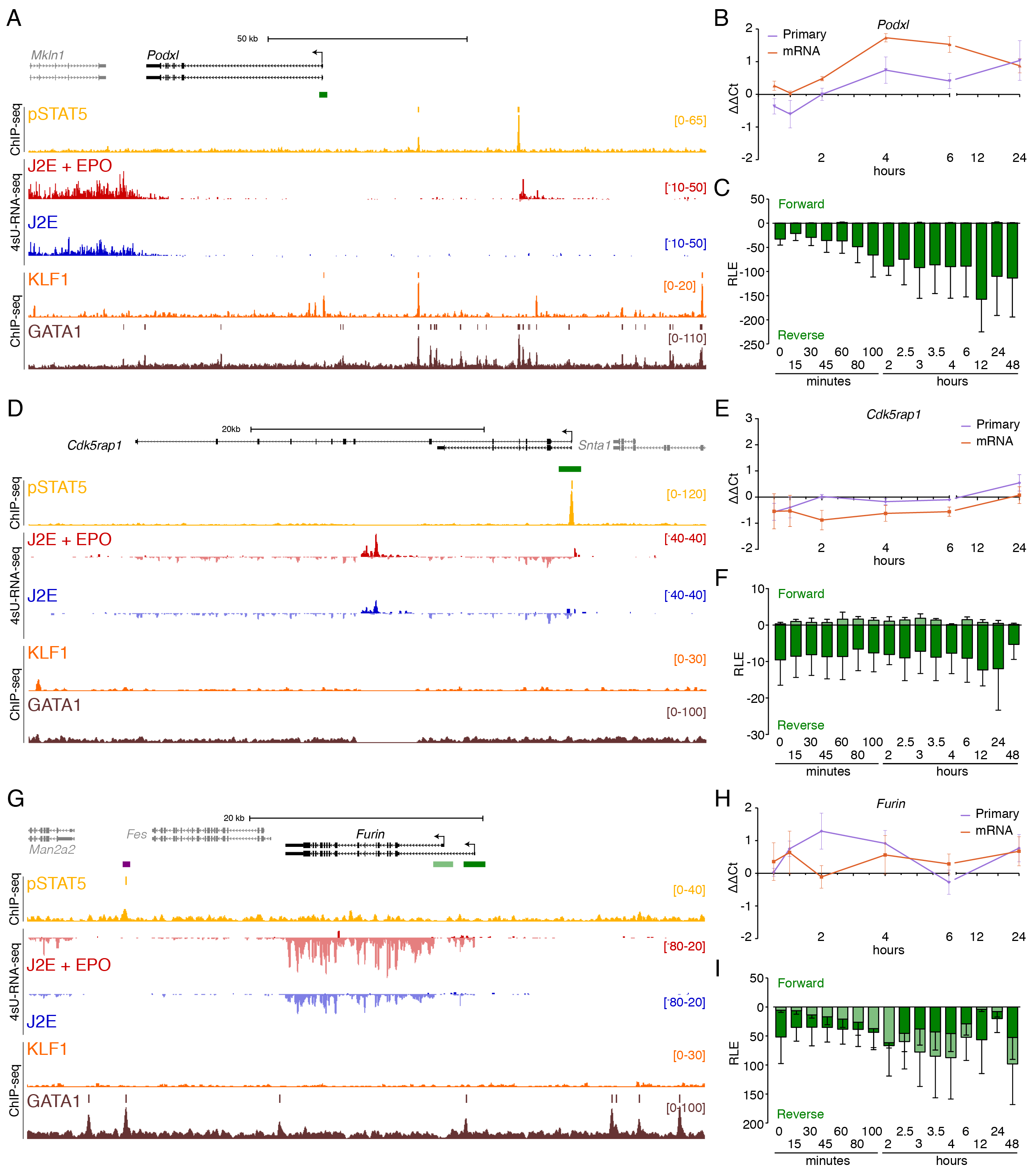

Supplement: S5 Fig — (A) ChIP-seq, 4sU-RNA-seq, qRT-PCR and CAGE tags from erythroid cells following 30 mins of EPO induction across the Podxl (A-C), Cdk5rap1 (D-F), and Furin (G-I) genes respectively. The overall design and colour coding of tracks is the same as for Fig 3. There are two strong pSTAT5 peaks 25kb and 50kb upstream of the Podxl TSS which overlap with GATA1 and KLF1 ChIP-seq peaks. However, Podxl is not upregulated by 4sU-RNA-seq (red track) after 30 mins, but shows weak and delayed upregulation of expression which is not significant until 4 hours in qRT-PCR and CAGE. There is a strong pSTAT5 peak at the Cdk5rap1 promoter but no significant upregulation in 4sU-RNA-seq (red track), qRT-PCR or CAGE following EPO induction. Significant upregulation of Furin transcription (red track) can be seen following EPO stimulation. Only a weak pSTAT5 peak can be seen downstream of Furin near to the neighbouring gene, Fes, but there are no other pSTAT5 peaks at the Furin promoter or within 30 kb of the Furin TSS. qRT-PCR for Furin primary pre-spliced transcripts shows dynamic upregulation peaking at 2 hours post EPO stimulation but processed mRNA is not substantially upregulated. CAGE tags at the two alternative Furin promoters show basal expression and delayed gradual upregulation from the second promoter (light green bar) until 4 hours post -EPO stimulation. (TIF) [file pone.0180922.s005.tif]

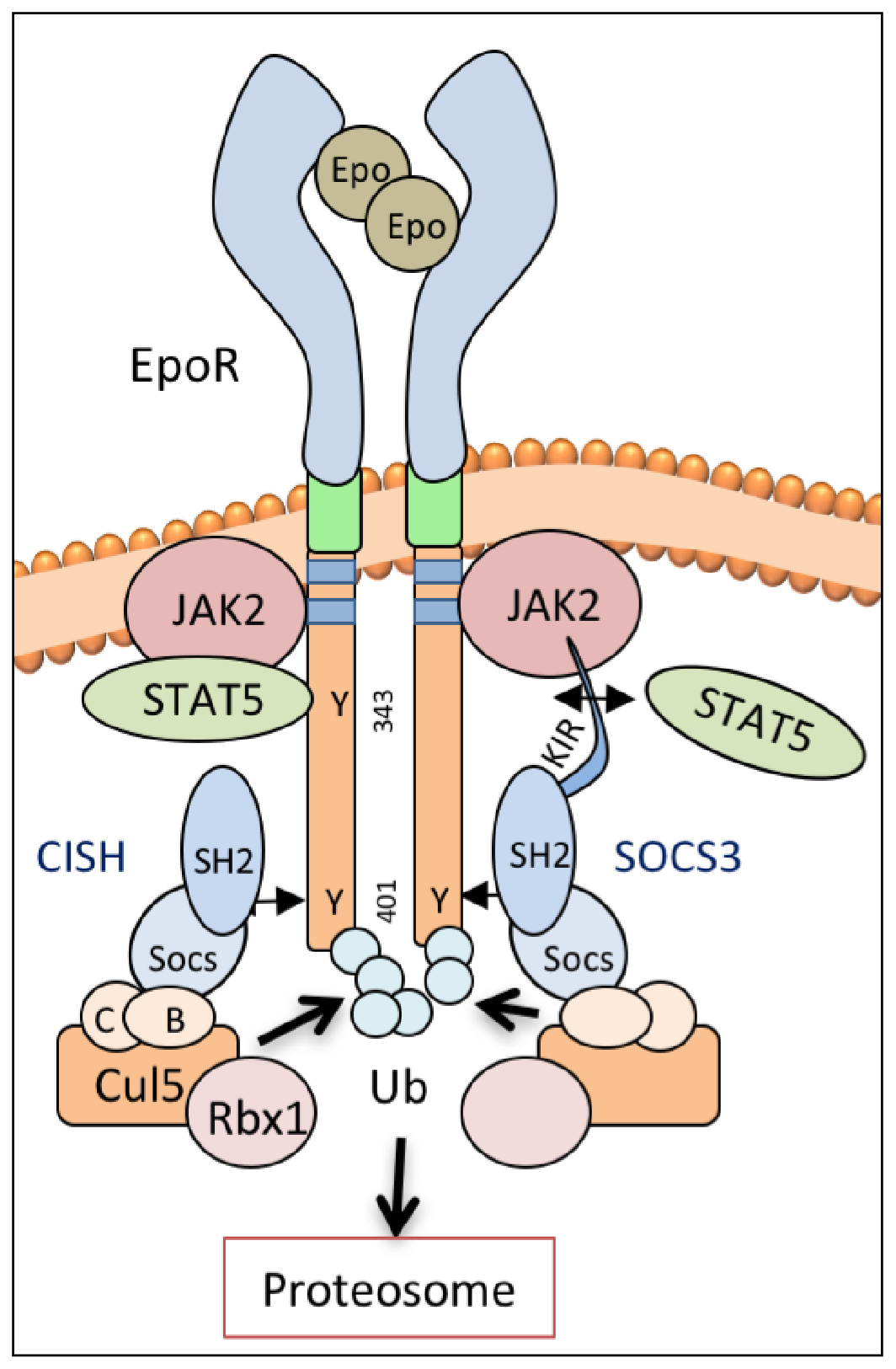

Supplement: S6 Fig — CISH binds activated pY401 in the EpoR via its SH2 domain. It recruits Elongin B via a C-terminal SOCS box and indirectly recruits Elongin C, which then recruits cullin-5 and Rbx1. This complex functions as an E3 ubiquitin ligase, to target EpoR and associated proteins for degradation in the proteasome. SOCS3 functions in a similar way via binding to pY401 but in addition it directly inhibits the kinase activity via competitive displacement of STAT5 binding in the active pocket of JAK2. It achieves this additional function via the N-terminal KIR domain which is not present in CISH. (TIF) [file pone.0180922.s006.tif]
