# Supplementary material for: Direct targets of pSTAT5 signalling in erythropoiesis
Source: PLoS One. 2017 Jul 21;12(7):e0180922. doi: 10.1371/journal.pone.0180922 (PMC5521770; doi:10.1371/journal.pone.0180922)
Supplement: S3 Table — (DOCX) [file pone.0180922.s009.docx]

Supplementary Table 1 - qPCR primers for ChIP validation.

| Primer Set | Target | Strand | Sequence (5′-3′) |
| --- | --- | --- | --- |
| Abcg2 5′ | *gDNA* | Forward  Reverse | ATGTGTCCAGCTGCTCCTG  AGAACTCGCACACACTCAGAC |
| Abcg2 GAS 3 | *gDNA* | Forward  Reverse | TTGTGACTTCCCTCTGATGGC  TATGAGCCAGGCCAGTTTCC |
| Abcg2 Middle | *gDNA* | Forward  Reverse | AGACACCCTGGTCTTACTTTGC  CTGAACCAGAGCAAAGAGTCAG |
| Abcg2 GAS 4 | *gDNA* | Forward  Reverse | TAAGTAGCCACCTTAGGGAGTG  CGGAGGAAGGGAGATCTGTATC |
| Abcg2 3′ | *gDNA* | Forward  Reverse | AATAGAATCTGGGGCCTGTGAG  AGTCTGGGTGGCCATCATATG |
| Bcl-x 5′ | *gDNA* | Forward  Reverse | ACATACTGCCACTGAGTACCAC  TGAGAAGAGCCCAGCCTAATTG |
| Bcl-x BS | *gDNA* | Forward  Reverse | TGAGCTTCAGGGAATCTTTGGG  CCGCTTCCTGTTCTGAGAAATG |
| Bcl-x 3′ | *gDNA* | Forward  Reverse | TTTGCATGAGGTCCTCCAAC  ACTCTGTGTGATCATGGCAGTC |
