# Supplementary material for: Direct targets of pSTAT5 signalling in erythropoiesis
Source: PLoS One. 2017 Jul 21;12(7):e0180922. doi: 10.1371/journal.pone.0180922 (PMC5521770; doi:10.1371/journal.pone.0180922)
Supplement: S4 Table — (DOCX) [file pone.0180922.s010.docx]

Supplementary Table 2 – qRT-PCR primers for gene expression validation.

| Gene ID | Target | Strand | Sequence (5′-3′) |
| --- | --- | --- | --- |
| *Hprt* | Primary | Forward  Reverse | ACGTAGGAGGACCCTTTAATGC TGCGCTCATCTTAGGCTTTG |
| *Hprt* | mRNA | Forward  Reverse | GCAGTACAGCCCCAAAATGG AACAAAGTCTGGCCTGTATCCAA |
| *Hbb* | Primary | Forward  Reverse | ACTTCTTGTGAGCTGCCTG  ACTGTGACAAGCTGCATGTG |
| *Hbb* | mRNA | Forward  Reverse | AAAGGTGAACGCCGATGAAG  ACTGTGACAAGCTGCATGTG |
| *Furin* | Primary | Forward  Reverse | tgttttctgcagccacatgc  TAGCCCAATCATCAGCGTTG |
| *Furin* | mRNA | Forward  Reverse | TTGGCAGCTGGTATCATTGC  TAGCCCAATCATCAGCGTTG |
| *Podxl* | Primary | Forward  Reverse | aagcctgggaatcacttgtg  AGTGCACAAATCCTCAGCTG |
| *Podxl* | mRNA | Forward  Reverse | AGTCAAAGCGTCCTTCAAGC  ATACACGGCCTTAGGAGAAAGC |
| *Poli* | Primary | Forward  Reverse | AGTGTTCCTCAGAAGTGGAAGC  tgatgaggcaaggtgaaacg |
| *Poli* | mRNA | Forward  Reverse | TGCAATTGCTCAGCGTATCC  AGCTTCCACTTCTGAGGAACAC |
| *Gypc* | Primary | Forward  Reverse | ttcctcaccgtgggtttttg  ttcttggctgctagaaaggc |
| *Gypc* | mRNA | Forward  Reverse | ACGGCATTATGGAGATTGCC  TGCAAACTCTGTGCCTTTGG |
| *Suv420h2* | Primary | Forward  Reverse | cacatgcctttgcccttttc  ACTTGTCTAGGGGCTTTGGTC |
| *Suv420h2* | mRNA | Forward  Reverse | TTCTATGGTGAGGGCTTCTTCG  TGGGCTGAAGTCTGAAAGCTC |
| *Socs3* | Primary | Forward  Reverse | AAGGCCGGAGATTTCGCTTC  aggagaaaccgggaaaagctc |
| *Socs3* | mRNA | Forward  Reverse | AGCCCCTTTGTAGACTTCACG  GGAAACTTGCTGTGGGTGAC |
| *N4bp2* | Primary | Forward  Reverse | tgtctctcccctggacttttc  ACTCAATGCGCTCGGTTTTC |
| *N4bp2* | mRNA | Forward  Reverse | AAACCGAGCGCATTGAGTTG  AAGGGTCTTCTCTTCCGTTAGC |
| *Cdk5rap1* | Primary | Forward  Reverse | atcttgtgacggcgaatgtg  GGCACGATACAGTAACTGCAC |
| *Cdk5rap1* | mRNA | Forward  Reverse | TGGCTTTTGTGGAGAGACAGAG  TCCTTCAGCCTGTGATATGCTC |
